# Sex-related differences in the associations between plasma free fatty acid levels and clinical features in patients with hypertrophic cardiomyopathy

**DOI:** 10.1186/s13293-016-0118-2

**Published:** 2016-11-25

**Authors:** Chengzhi Yang, Changlin Zhang, Jiansong Yuan, Jingang Cui, Shengwen Liu, Fenghuan Hu, Weixian Yang, Xuanye Bi, Shubin Qiao

**Affiliations:** State Key Laboratory of Cardiovascular Disease, Fuwai Hospital, National Center for Cardiovascular Diseases, Chinese Academy of Medical Sciences and Peking Union Medical College, Beijing, 100037 China

**Keywords:** Hypertrophic cardiomyopathy, Free fatty acid, Sex, Left ventricular mass index, Left atrium diameter

## Abstract

**Background:**

Previous studies have indicated that inefficient energy utilization may play a pivotal role in hypertrophic cardiomyopathy (HCM). However, whether plasma free fatty acid (FFA), a main energy substrate of heart, has an effect on HCM remains unclear. Besides, several studies have suggested sex-related differences in HCM features and FFA metabolism. Here, we aimed to explore the association between plasma FFA levels and HCM and potential effects of sex on this relation.

**Methods:**

A total of 412 patients (age 47.8 ± 12.7 years, 243 males (59.0%)) with HCM were recruited. Complete medical history was collected. Echocardiography and cardiovascular magnetic resonance imaging (CMRI) were performed. Fasting plasma FFA was determined by clinical laboratory. Left ventricular mass (LVM), maximum wall thickness (MWT), and left atrium diameter (LAD) were assessed with CMRI.

**Results:**

The median FFA levels were 0.38 (interquartile range (IQR) 0.27–0.52) mmol/L in men and 0.40 (IQR 0.30–0.59) mmol/L in women. The FFA levels were significantly lower in men compared with those in women (*p* = 0.005). Compared with women, men had greater LVM index (LVMI) (96.8 ± 37.6 vs. 78.6 ± 31.5 g/m^2^, *p* < 0.001). FFA levels in male patients correlated positively with LVM, LVMI, LAD, cholesterol levels, high-density lipoprotein-cholesterol (HDL-C) levels, heart rate, and systolic blood pressure (SBP). However, none of these variables were significantly associated with sqrt (FFA) in female patients except a borderline correlation of LAD (*p* = 0.050). Multiple linear regression analysis was performed in male patients and revealed that HDL-C (*β* = 0.191, *p* = 0.002), heart rate (*β* = 0.182, *p* = 0.004), SBP (*β* = 0.167, *p* = 0.007), LVMI (*β* = 0.132, *p* = 0.032), and LAD (*β* = 0.165, *p* = 0.009) were independently associated with increasing FFA levels.

**Conclusions:**

In patients with HCM, LVMI, LAD, HDL-C, SBP, and heart rate were independently associated with increasing plasma FFA levels in males, whereas not in females. These results suggest that sex may affect the pathogenesis of HCM through influencing FFA metabolism. And these sex-related differences should be taken into account in therapeutic approaches to influence myocardial FFA metabolism in HCM.

## Background

Hypertrophic cardiomyopathy (HCM), a common cause of sudden death in young people, is characterized by primary asymmetric left ventricular hypertrophy without an alternative cause [1, 2]. With a prevalence of 2‰ in the general population, HCM has been established as the most frequent genetic heart disease [3, 4]. More than 1400 mutation sites in at least 11 genes encoding proteins of the cardiac sarcomere are responsible for (or associated with) HCM [3–6]. However, pathways from mutation to clinical phenotype are still poorly understood [2]. Nevertheless, some studies have showed sex differences in clinical features. Male patients had greater left ventricular mass index (LVMI) [7] and disease penetrance [8], while female patients were more likely to have severe symptoms of heart failure [9].

Several studies have suggested that inefficient energy utilization resulted from sarcomeric mutations plays a pivotal role in HCM [2, 3]. Circulating free fatty acids (FFAs) are mainly from lipolysis in the adipose tissue [10]. It is well known that FFAs are important energy substrates of the heart, where FFA oxidation supplies about 70% of energy consumption [10, 11]. However, high concentrations of FFA under pathological conditions have been shown to be proarrhythmic [12] and exacerbate heart failure [13, 14], which are common clinical features of HCM. Besides, many studies indicate that pathologic hypertrophy [11, 15] and heart failure [13, 16] are associated with a reduction in FFA oxidation. In addition, previous data suggest that reduced myocardial long-chain FFA uptake contributes to some types of HCM [17]. Moreover, septal hypertrophy is a feature of the developing mammalian heart [18], where FFA utilization is limited [19]. Early infants (less than 6 months of age) in whom if the shift of myocardial substrate utilization from glucose to FFA is prevented sometimes exhibit typical features of HCM [20]. Therefore, FFA may play an important role in HCM. Furthermore, recent literature has suggested that female patients with heart failure had higher myocardial FFA uptake [21]. And there were sex-related differences in serum FFA levels [22] and FFA metabolism [23].

However, to date, few studies have been performed to evaluate the associations of FFA with cardiac structural and functional parameters. Hence, here we sought to explore the relation between FFA levels and HCM with a large cohort of HCM patients. Considering aforementioned sex-related differences in HCM features and FFA metabolism, we determined potential effects of sex on the relation between FFA and HCM. Owing to its three-dimensional tomographic imaging with high spatial resolution, cardiac magnetic resonance imaging (CMRI) was employed in the diagnosis and morphological characterization of HCM in the present study [24].

## Methods

### Study population

The protocol of this study was approved by Fuwai Hospital (Beijing, China) ethics committee and complied with the Declaration of Helsinki. The informed consents were obtained from all participants.

Consecutive patients with HCM, evaluated at Fuwai Hospital (Beijing, China) from December 2012 to December 2015, were enrolled. The diagnosis of HCM was based on a maximum left ventricular wall thickness ≥15 mm (or ≥13 mm with an unequivocal family history of HCM), as measured by echocardiography or CMRI, in the absence of other cardiac or systemic diseases capable of producing such magnitude of hypertrophy [25]. Patients were excluded if they had valvular heart disease, significant coronary artery disease (epicardial coronary stenosis >70% on coronary angiography, previous myocardial infarction, bypass surgery, or percutaneous coronary intervention), diabetes mellitus, infection, renal dysfunction (defined as an estimated glomerular filtration rate <60 mL/min/1.73 m^2^), concomitant neoplasma, connective tissue disease, and pregnancy. Finally, a total of 412 patients with HCM were recruited in the present study. Relevant clinical variables including complete medical history, physical examination, 12-lead electrocardiography, 24-h ambulatory electrocardiographic monitoring, blood examination, transthoracic echocardiography, and CMRI were collected.

### Echocardiography

All transthoracic echocardiography were performed by experienced cardiologists as recommended by the American Society of Echocardiography [26], using an iE33 Color Doppler Ultrasound System (Philips Healthcare, Andover, MA). Two-dimensional, M-mode images and Doppler tracings were obtained. The peak velocity across the left ventricular outflow tract (LVOT) was also measured, and the peak pressure gradient was estimated using the simplified Bernoulli equation. The presence of LVOT obstruction was defined as an instantaneous peak Doppler LVOT gradient ≥30 mmHg at rest or during physiological provocation, such as Valsalva maneuver, standing, and exercise. The provoked LVOT gradient was only measured in patients with a LVOT gradient <50 mmHg at rest.

### CMRI

CMRI was performed using a 1.5-T speed clinical scanner (Magnetom Avanto; Siemens Medical Solutions, Erlangen, Germany). The imaging protocol and analysis have been described previously [27]. Briefly, a true fast imaging with steady-state precession (TrueFISP) sequence was used to obtain cine images. Full ventricular coverage was achieved with 6-mm-thick slices. CMR images were analyzed by an experienced radiologist with a workstation (Siemens Medical Systems, Erlangen, Germany). Endocardial and epicardial contours of the left ventricular (LV) myocardium (excluding papillary muscles) were manually traced at end-diastole and end-systole on each LV short-axis cine image. LV end-diastolic volume (LVEDV), LV end-systolic volume (LVESV), left ventricular ejection fraction (LVEF), stroke volume, cardiac output, and LV mass (LVM) were then calculated in a standard fashion. LVM was derived by multiplying LV myocardial volume measured at end-diastole with the specific gravity of myocardium (1.05 g/mL). All those parameters were indexed to body surface area, except LVEF. The LVED diameter and maximal LV wall thickness were traced and measured from the short-axis views at end-diastole.

### Laboratory measurements

Fasting blood samples were collected for all enrolled subjects within 2 days of TTE and 1 week of CMRI examination. Then the laboratory measurements were conducted by the clinical laboratory. All assays were performed within 4 h of blood collection by medical technologists who were unaware of any clinical information about the studied patients. Plasma FFA was measured with enzymatic assay (DiaSys Diagnostic Systems GmbH, Germany) on a Beckman-Coulter LX20. The intra-assay coefficient of variation was 1.03%, and the inter-assay coefficient of variation was 1.11%. Serum levels of triglycerides, high-density lipoprotein-cholesterol (HDL-C), and low-density lipoprotein-cholesterol (LDL-C) were measured by routine laboratory methods using an Olympus AU-5400 auto-analyzer (Olympus Corporation, Mishama, Japan). Plasma levels of N-terminal proB-type natriuretic peptide (NT-proBNP) were measured using an electrochemiluminescent immunoassay (Elecsys proBNP II assay; Roche Diagnostics, Mannheim, Germany). Hyperlipidemia was defined as those with serum LDL-C ≥3.37 mmol/L or triglycerides ≥1.70 mmol/L.

### Statistical analysis

The values were expressed as mean ± SD or median (interquartile range (IQR)) for the continuous variables and as the number (percentage) for the categorical variables. Comparisons of continuous variables between two groups were assessed using independent Student’s *t* test or Mann-Whitney *U* test depending on the distribution of variables. Categorical variables were compared with *χ* ^2^ test, and Fisher’s exact test was used when the expected frequency was <5. Pearson’s correlation test or Spearman’s correlation test was used to examine correlations between two continuous variables when indicated. Stepwise multiple linear regression analysis (*p* value threshold to enter 0.05; to remove, 0.10) was conducted to identify independent variables that might determine FFA levels. In order to obtain normal distribution, square root transformation was applied to plasma FFA in correlation tests and multiple linear regression analysis. Variables with a *p* value <0.10 in the univariate analysis were included in the multiple regression analysis. A two-tailed *p* value <0.05 was considered as statistically significant. Statistical analysis was performed with SPSS version 19.0 software (SPSS Inc., Chicago, IL).

## Results

**Fig. 1:**
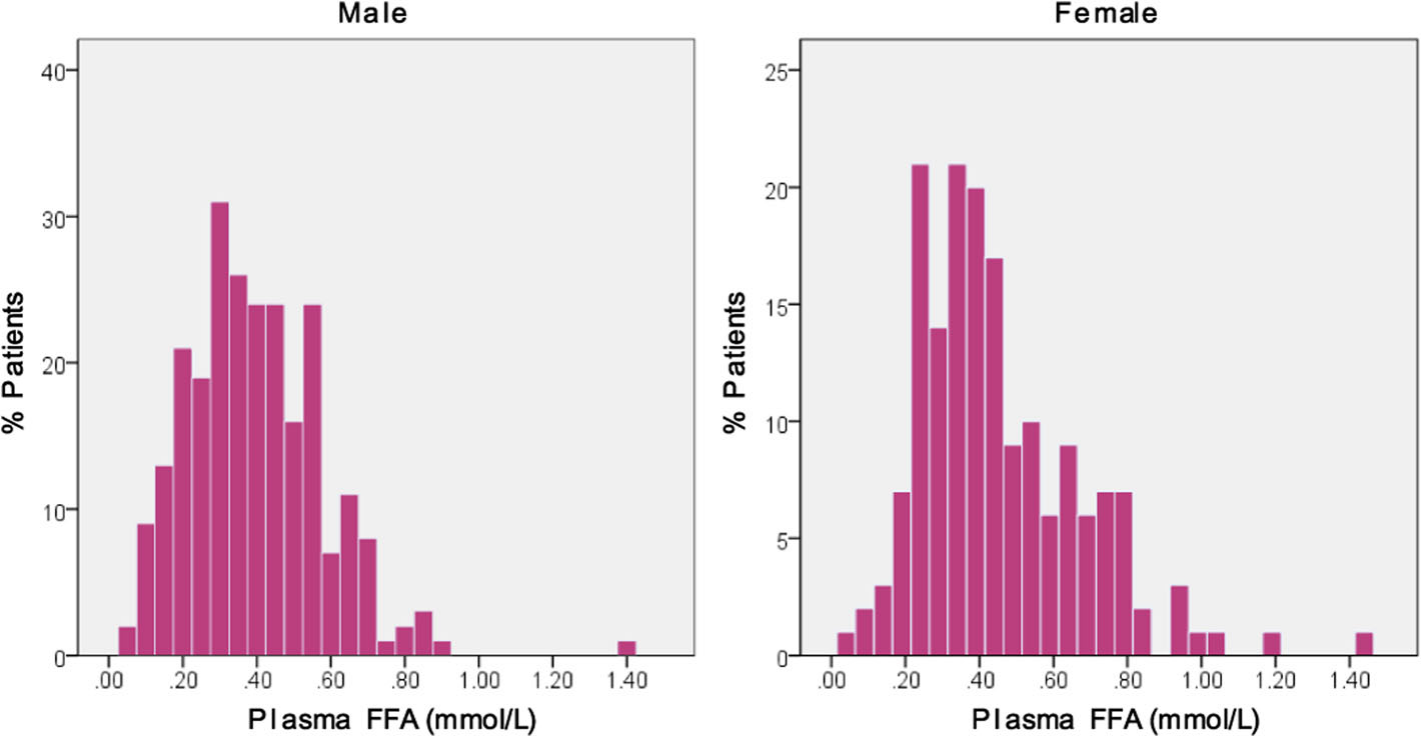
Frequency distribution of plasma FFA levels in male and female patients with hypertrophic cardiomyopathy

The clinical characteristics of studied population stratified by sex are summarized in Table 1. A total of 412 HCM patients were enrolled in the present study, comprised of 243 males (59.0%) and 169 females (41.0%). As shown in Fig. 1, the median FFA levels were 0.38 (0.27–0.52) mmol/L in men and 0.40 (0.30–0.59) mmol/L in women. The FFA levels were significantly lower in men compared with those in women (*p* = 0.005). Besides, men were younger (45.6 ± 11.9 vs. 51.0 ± 13.0 years, *p* < 0.001) than women. There were more patients with maximum wall thickness ≥30 mm and resting LVOTG ≥30 mmHg in men. In contrast, men had lower NT-pro-BNP levels. Of note, serum HDL-C levels were significantly lower in men (1.06 ± 0.30 vs. 1.27 ± 0.33 mmol/L, *p* < 0.001). Additionally, men had higher BMI and diastolic blood pressure and were more likely to smoke (56.8 vs. 3.0%, *p* < 0.001).

**Table 1:** Clinical characteristics of patients with hypertrophic cardiomyopathy stratified by sex

Variable	Male (*n* = 243)	Female (*n* = 169)	*p* value
FFAs (mmol/L)	0.40 ± 0.18	0.45 ± 0.22	0.005
Age (years)	45.6 ± 11.9	51.0 ± 13.0	<0.001
BMI (kg/m^2^)	25.7 ± 3.2	24.6 ± 3.4	0.001
Systolic blood pressure (mmHg)	119.0 ± 16.0	116.1 ± 18.0	0.088
Diastolic blood pressure (mmHg)	70.0 (68.0–80.0)	70.0 (60.0–80.0)	0.026
Heart rate (beats/min)	70.0 (65.0–80.0)	70.0 (64.0–77.0)	0.073
NYHA functional class III or IV, *n* (%)	80 (32.9%)	65 (38.5%)	0.251
Chest pain, *n* (%)	107 (44.0%)	79 (46.7%)	0.615
Palpitation, *n* (%)	72 (29.6%)	63 (37.3%)	0.110
Family history of HCM, *n* (%)	36 (14.8%)	20 (11.8%)	0.465
Atrial fibrillation, *n* (%)	29 (11.9%)	25 (14.8%)	0.458
Risk factors for SCD			
Family history of SCD, *n* (%)	12 (4.9%)	8 (4.7%)	1.000
Syncope, *n* (%)	64 (26.3%)	46 (27.2%)	0.910
Maximum wall thickness ≥30 mm, *n* (%)	55 (22.6%)	18 (10.7%)	0.002
Resting LVOTG ≥30 mmHg, *n* (%)	188 (79.7%)	147 (90.2%)	0.005
Non-sustained VT^a^, *n* (%)	20 (12.6%)	18 (16.1%)	0.478
Cardiovascular risk			
Hypertension, *n* (%)	70 (28.8%)	59 (34.9%)	0.197
Hyperlipidemia, *n* (%)	76 (31.3%)	54 (32.0%)	0.914
Current smokers, *n* (%)	138 (56.8%)	5 (3.0%)	<0.001
Laboratory test			
Cholesterol (mmol/L)	4.43 ± 0.99	4.63 ± 0.97	0.042
LDL-C (mmol/L)	2.83 ± 0.86	2.89 ± 0.82	0.502
HDL-C (mmol/L)	1.06 ± 0.30	1.27 ± 0.33	<0.001
Triglycerides (mmol/L)	1.37 (1.00–1.97)	1.27 (0.95–1.77)	0.067
NT-pro-BNP (pmol/L)	962 (518–1656)	1605 (956–2664)	<0.001
Medications			
β-Blockers, *n* (%)	182 (74.9%)	124 (73.4%)	0.732
Non-dihydropyridine CCB, *n* (%)	31 (12.8%)	32 (18.9%)	0.096
Dihydropyridine CCB, *n* (%)	10 (4.1%)	9 (5.3%)	0.364
ACEI/ARB, *n* (%)	27 (11.1%)	19 (11.2%)	1.000
Aspirin, *n* (%)	42 (17.3%)	32 (18.9%)	0.697
Statins, *n* (%)	26 (10.7%)	26 (15.7%)	0.176
Diuretics, *n* (%)	6 (2.5%)	16 (9.5%)	0.003
Trimetazidine, *n* (%)	10 (4.1%)	4 (2.4%)	0.415

Data are expressed as mean ± SD, median (interquartile range), or number (percentage)

*ACEI* angiotensin-converting enzyme inhibitor, *ARB* angiotensin receptor blocker, *BMI* body mass index, *CCB* calcium channel blocker, *HCM* hypertrophic cardiomyopathy, *LDL-C* low-density lipoprotein-cholesterol, *HDL-C* high-density lipoprotein-cholesterol, *LVOTG* left ventricular outflow tract gradient, *NT-proBNP* N-terminal pro-B-type natriuretic peptide, *NYHA* New York Heart Association, *SCD* sudden cardiac death, *VT* ventricular tachycardia

^a^Ambulatory 24-h Holter monitoring data were obtained in 195 of the 412 study patients

Echocardiographic and cardiovascular magnetic resonance imaging data of the patients with HCM are presented in Table 2. The left ventricular outflow tract gradient (LVOTG) levels at rest were lower in men than those in women. However, men had greater LVOTG after provocation. Compared with women, men also had greater LV end-diastolic diameter (*p* < 0.001) and maximum wall thickness (*p* < 0.001). In addition, in comparison with women, men had greater LV end-diastolic volume, LV end-systolic volume, stroke volume, cardiac output, and LV mass. However, after being adjusted with body area, only LV mass index (LVMI) was still higher in men (96.8 ± 37.6 vs. 78.6 ± 31.5 g/m^2^, *p* < 0.001).

**Table 2:** Echocardiographic and cardiovascular magnetic resonance data of male and female patients with hypertrophic cardiomyopathy

Variable	Male (*n* = 243)	Female (*n* = 169)		*p* value	
Echocardiography					
Systolic anterior motion, *n* (%)	215 (88.5%)		156 (92.3%)		0.242
LVOT obstruction, *n* (%)	241 (99.2%)		168 (99.4%)		1.000
Severe mitral regurgitation, *n* (%)	13 (5.3%)		11 (6.5%)		0.672
LVOTG at rest (mmHg)	64.3 ± 35.4		79.6 ± 35.9		<0.001
LVOTG after provocation (mmHg)^a^	82.1 ± 31.4		66.3 ± 28.7		0.031
Cardiovascular magnetic resonance					
Left atrium diameter (mm)	42.1 ± 8.1		42.8 ± 8.1		0.362
LV end-diastolic diameter (mm)	46.8 ± 5.0		45.0 ± 5.2		<0.001
Maximum wall thickness (mm)	25.3 ± 5.5		23.4 ± 4.9		<0.001
LV ejection fraction (%)	67.8 ± 8.0		69.1 ± 7.6		0.097
LV end-diastolic volume (mL)	135.0 ± 34.4		114.3 ± 27.1		<0.001
LV end-systolic volume (mL)	44.7 ± 18.9		36.4 ± 15.4		<0.001
Stroke volume (mL)	91.1 ± 23.6		78.4 ± 17.6		<0.001
Cardiac output (L/min)	6.3 ± 1.8		5.3 ± 1.3		<0.001
LV mass (g)	181.3 ± 73.7		128.6 ± 54.2		<0.001
LV end-diastolic volume index (mL/m^2^)	72.5 ± 16.4		70.1 ± 15.2		0.140
LV end-systolic volume index (mL/m^2^)	23.7 ± 9.3		22.0 ± 8.7		0.075
Stroke volume index (mL/m^2^)	48.9 ± 11.3		48.1 ± 9.9		0.502
Cardiac index (L/min/m^2^)	3.4 ± 0.9		3.3 ± 0.8		0.306
LV mass index (g/m^2^)	96.8 ± 37.6		78.6 ± 31.5		<0.001

Data are expressed as mean ± SD or number (percentage)

*HCM* hypertrophic cardiomyopathy, *LV* left ventricular, *LVOTG* left ventricular outflow tract gradient

^a^Provoked LVOTG data were available in 96 patients

Table 3 shows plasma FFA levels with respect to clinical characteristics of the male and female patients with HCM. In men, patients with resting LVOTG ≥30 mmHg had higher FFA levels (*p* = 0.028). In women, greater FFA levels were found in those who had palpitation (*p* = 0.025), non-sustained ventricular tachycardia (*p* = 0.015), and hyperlipidemia (*p* = 0.040). Notably, smoking and statin therapy did not affect FFA levels both in men and women.

**Table 3:** Plasma FFA levels with respect to clinical characteristics of the male and female patients with HCM

Variable	FFA levels (mmol/L) in male patients		FFA levels (mmol/L) in female patients			
	Present	Absent	*p* value	Present	Absent	*p* value
NYHA functional class III or IV	0.36 (0.27–0.53)	0.38 (0.27–0.52)	0.754	0.42 (0.29–0.57)	0.39 (0.30–0.61)	0.649
Chest pain	0.38 (0.29–0.53)	0.37 (0.26–0.57)	0.346	0.42 (0.31–0.57)	0.38 (0.30–0.59)	0.521
Palpitation	0.40 (0.27–0.49)	0.36 (0.27–0.53)	0.560	0.49 (0.34–0.69)	0.38 (0.29–0.51)	0.025
Family history of HCM	0.35 (0.25–0.44)	0.38 (0.27–0.53)	0.148	0.42 (0.29–0.46)	0.39 (0.30–0.62)	0.626
Atrial fibrillation	0.46 (0.37–0.54)	0.36 (0.27–0.52)	0.053	0.42 (0.36–0.59)	0.39 (0.29–0.59)	0.285
Risk factors for SCD						
Family history of SCD	0.28 (0.14–0.34)	0.38 (0.27–0.52)	0.091	0.42 (0.21–0.60)	0.40 (0.30–0.59)	0.904
Syncope	0.36 (0.22–0.51)	0.39 (0.28–0.52)	0.120	0.36 (0.25–0.52)	0.42 (0.32–0.61)	0.170
Maximum wall thickness ≥30 mm	0.39 (0.28–0.54)	0.37 (0.27–0.50)	0.111	0.42 (0.25–0.68)	0.40 (0.30–0.56)	0.700
Resting LVOTG ≥30 mmHg	0.39 (0.28–0.53)	0.30 (0.22–0.47)	0.028	0.40 (0.30–0.58)	0.45 (0.35–0.62)	0.393
Non-sustained VT^a^	0.37 (0.30–0.56)	0.37 (0.27–0.50)	0.533	0.51 (0.37–0.65)	0.37 (0.25–0.50)	0.015
Cardiovascular risk						
Hypertension	0.40 (0.29–0.50)	0.36 (0.27–0.53)	0.424	0.39 (0.30–0.63)	0.40 (0.30–0.56)	0.692
Hyperlipidemia	0.40 (0.28–0.53)	0.36 (0.27–0.51)	0.257	0.44 (0.35–0.64)	0.38 (0.27–0.54)	0.040
Smoking	0.36 (0.24–0.50)	0.39 (0.28–0.54)	0.094	0.30 (0.24–0.60)	0.40 (0.30–0.59)	0.492
Medications						
β-Blockers	0.37 (0.26–0.50)	0.40 (0.30–0.56)	0.054	0.39 (0.30–0.57)	0.43 (0.32–0.66)	0.321
Non-dihydropyridine CCB	0.38 (0.28–0.48)	0.38 (0.27–0.52)	0.652	0.42 (0.36–0.55)	0.39 (0.30–0.61)	0.626
Dihydropyridine CCB	0.38 (0.30–0.44)	0.38 (0.27–0.53)	0.790	0.30 (0.26–0.53)	0.41 (0.30–0.61)	0.238
ACEI/ARB	0.39 (0.31–0.47)	0.38 (0.26–0.52)	0.533	0.38 (0.34–0.55)	0.40 (0.30–0.59)	0.739
Aspirin	0.40 (0.30–0.55)	0.37 (0.26–0.51)	0.112	0.43 (0.31–0.56)	0.39 (0.30–0.60)	0.617
Statins	0.42 (0.33–0.55)	0.36 (0.27–0.51)	0.119	0.51 (0.33–0.62)	0.39 (0.30–0.56)	0.175
Diuretics	0.40 (0.33–0.67)	0.37 (0.27–0.52)	0.273	0.37 (0.24–0.56)	0.40 (0.30–0.60)	0.393
Trimetazidine	0.38 (0.26–0.50)	0.37 (0.27–0.50)	0.940	0.29 (0.14–0.41)	0.40 (0.30–0.59)	0.097
Echocardiography						
Systolic anterior motion	0.38 (0.27–0.51)	0.39 (0.29–0.56)	0.580	0.41 (0.30–0.59)	0.34 (0.23–0.55)	0.292
Severe mitral regurgitation	0.34 (0.25–0.52)	0.38 (0.27–0.52)	0.816	0.57 (0.40–0.73)	0.39 (0.30–0.56)	0.054

Data are expressed as median (interquartile range)

*ACEI* angiotensin-converting enzyme inhibitor, *ARB* angiotensin receptor blocker, *CCB* calcium channel blocker, *HCM* hypertrophic cardiomyopathy, *LVOTG* left ventricular outflow tract gradient, *NT-proBNP* N-terminal pro-B-type natriuretic peptide, *NYHA* New York Heart Association, *SCD* sudden cardiac death.

^a^Ambulatory 24-h Holter monitoring data were obtained in 195 of the 412 study patients

**Fig. 2:**
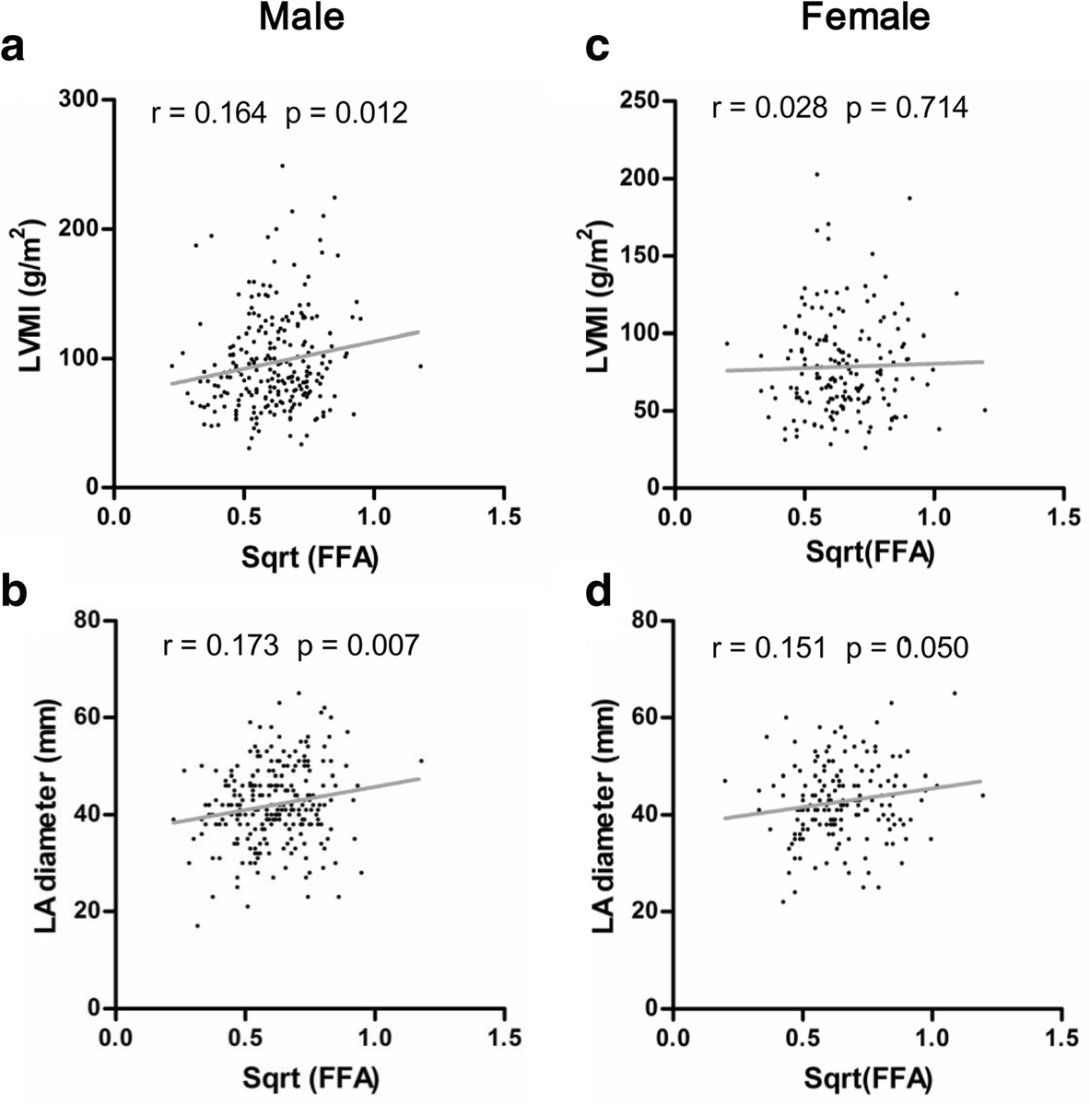
Sex differences in relation of sqrt (FFA) with left ventricular mass index (LVMI) and left atrium diameter (LAD). **a**, **b** Correlations between sqrt (FFA) and LVMI and LA diameter in male patients with hypertrophic cardiomyopathy (HCM). **c**, **d** Correlations between sqrt (FFA) and LVMI and LA diameter in female patients with (HCM)

**Fig. 3:**
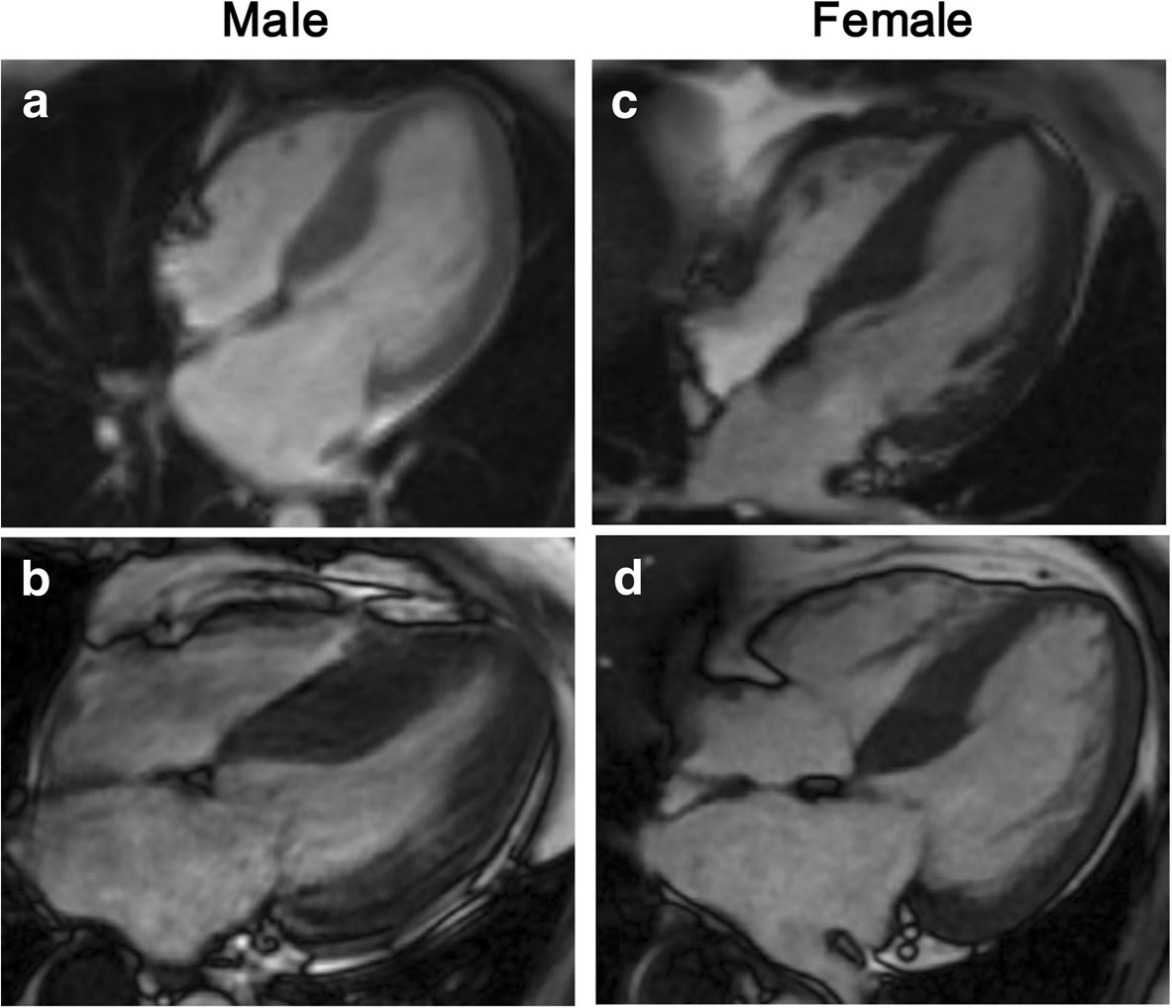
Representative CMRI images indicating the correlations between FFA and left ventricular mass index (LVMI) in male and female patients with hypertrophic cardiomyopathy. **a**, **b** End-diastolic four-chamber view cine images of two male patients with plasma FFA levels of 0.22 and 0.69 mmol/L, and their LVIMI were 53.5 and 119.6 kg/m^2^, respectively. **c**, **d** Images of two female patients with plasma FFA levels of 0.30 and 0.73 mmol/L, and their LVIMI were 74.1 and 70.5 kg/m^2^, respectively

Univariate analysis of correlation between variables and FFA is presented in Table 4. Square root transformation was applied to plasma FFA (sqrt [FFA]) to abstain normal distribution. Sqrt (FFA) in male patients correlated positively with serum cholesterol (*r* = 0.134, *p* = 0.037), HDL-C (*r* = 0.138, *p* = 0.031), heart rate (*r* = 0.225, *p* < 0.001), and systolic blood pressure (SBP; *r* = 0.142, *p* = 0.028). Furthermore, there were also significant correlations between sqrt (FFA) and maximum wall thickness (MWT) (*r* = 0.169, *p* = 0.008), LVM (*r* = 0.161, *p* = 0.013), LVMI (*r* = 0.164, *p* = 0.012; Fig. 2a), and left atrium diameter (LAD) (*r* = 0.173, *p* = 0.007; Fig. 2b). However, none of these variables were significantly associated with sqrt (FFA) in female patients except a borderline correlation between sqrt (FFA) and LAD (*r* = 0.151, *p* = 0.050; Fig. 2c, d). The representative CMRI images indicating the correlations between sqrt (FFA) and LVMI and LAD are shown in Fig. 3.

**Table 4:** Univariate analysis of correlation between variables and sqrt (FFA) in male and female HCM patients

Variable	Male (*n* = 243)		Female (*n* = 169)	
	Correlation coefficient (*r*)	*p* value	Correlation coefficient (*r*)	*p* value
Age (years)	0.043	0.508	0.103	0.181
BMI (kg/m^2^)	0.047	0.463	0.050	0.516
Cholesterol (mmol/L)	0.134	0.037	0.020	0.799
LDL-C (mmol/L)	0.174	0.088	0.020	0.798
HDL-C (mmol/L)	0.138	0.031	0.045	0.560
Triglycerides (mmol/L)	0.066	0.307	−0.052	0.500
Systolic blood pressure (mmHg)	0.142	0.028	0.012	0.874
Heart rate (beats/min)	0.225	<0.001	0.120	0.123
NT-pro-BNP (pmol/L)	0.080	0.266	−0.013	0.871
Left atrium diameter (mm)	0.173	0.007	0.151	0.050
Maximum wall thickness (mm)	0.169	0.008	−0.053	0.495
LV end-diastolic diameter (mm)	−0.043	0.507	0.020	0.798
LV ejection fraction (%)	−0.026	0.689	−0.028	0.722
LV end-diastolic volume (mL)	0.060	0.353	0.101	0.192
LV end-systolic volume (mL)	0.099	0.127	0.076	0.137
Stroke volume (mL)	0.052	0.420	0.077	0.323
Cardiac output (L/min)	0.081	0.211	0.075	0.333
LV mass (g)	0.161	0.013	0.061	0.435
LV end-diastolic volume index (mL/m^2^)	0.083	0.204	0.070	0.366
LV end-systolic volume index (mL/m^2^)	0.058	0.371	0.070	0.370
Stroke volume index (mL/m^2^)	0.280	0.070	0.046	0.550
LV mass index (g/m^2^)	0.164	0.012	0.028	0.714

Abbreviations as in Tables 1 and 2

Then multiple linear regression analysis was performed to identify independent determinants of FFA levels in male patients (Table 5). HDL-C (*β* = 0.191, *p* = 0.002), heart rate (*β* = 0.182, *p* = 0.004), SBP (*β* = 0.167, *p* = 0.007), LVMI (*β* = 0.132, *p* = 0.032), and LAD (*β* = 0.165, *p* = 0.009) were independently associated with increasing sqrt (FFA).

**Table 5:** Multiple linear regression analysis for the association between sqrt (FFA) and variables in male HCM patients

Variables	Standardized coefficients (*β*)	*p* value
HDL-C (mmol/L)	0.191	0.002
Heart rate (beats/min)	0.182	0.004
Systolic blood pressure (mmHg)	0.167	0.007
LV mass index (g/m^2^)	0.132	0.032
Left atrium diameter (mm)	0.165	0.009

Given that LV mass was significantly associated with LV mass index, LV mass was not included in multiple linear regression analysis. Abbreviations as in Table 1. Multiple *R* = 0.382, *R* ^2^ = 0.146

## Discussion

Although many studies have demonstrated that HCM is mainly caused by mutations in genes encoding for the cardiac sarcomere in the past 20 years, mechanisms that lead from a sarcomeric mutation to diverse disease phenotypes remain unclear [2, 3]. Recent evidence has emerged that impaired myocardial energy metabolism may be a primary stimulus for hypertrophy in HCM [28, 29]. It has long been recognized that FFA is the main energy substrate of the heart. However, few studies have been performed to unravel the correlation between plasma FFA levels and HCM. For the first time, the current study revealed that LVMI, LAD, SBP, heart rate (HR), and HDL-C were independently and positively associated with increasing plasma FFA levels in male HCM patients. In contrast, none of these variables were significantly associated with FFA levels in female patients, suggesting sex-related differences in FFA effects on HCM.

Plasma FFAs originate primarily from adipose tissue lipolysis and serve as physiologically important energy substrates, especially for adult heart [10, 30]. In contrast, it has been reported that glucose, instead of FFA, is the major energy substrate for fetal heart [31]. Maron and Verter observed that asymmetric septal hypertrophy was a feature of developing mammalian hearts [18]. A shift of myocardial energy substrate utilization from glucose to FFA may be one signal that transforms fetal myocardium to mature adult myocardium [32]. Besides, prevention of this energy substrate shift may lead to typical features of HCM in early infants [33]. And some types of HCM may result from reduced uptake of myocardial long-chain fatty acid (LCFA) [17, 34]. Tuunanen et al. revealed that LV mass was inversely related to myocardial FFA uptake [35].

In the present study, we found that MWT, LVM, LVMI, and LAD, which reflect the severity of HCM, were significantly associated with plasma FFA levels in male patients on univariate analysis. After adjusting for other variables, LVMI and LAD were still independently associated with FFA levels. Since asymmetric left ventricular hypertrophy is the primary pathological feature of HCM, together with studies mentioned above, our findings indicate that FFA might play an important role in the pathogenesis of HCM.

Interestingly, these correlations were found only in male patients, indicating that sex may have some effects on the pathogenesis of hypertrophy in HCM. In fact, there is growing evidence that many genes are expressed/function in a “sexually dimorphic manner” [36–38]. Sex has been found to be an important modifying factor in HCM phenotype. On the one hand, male sex is predisposed to have greater LV mass [7]. On the other hand, Chen et al. showed that estrogen might prevent myocardial energy dysregulation in the mice model of HCM [39]. Additionally, recent literature suggested that women had higher FFA uptake and metabolism than men in nonischemic heart failure [21]. Therefore, we speculate that female sex may have protective effects on the pathogenesis of HCM.

Some studies suggested that high circulating FFA levels were associated with heart failure, pathologic hypertrophy, and sudden cardiac death (SCD) [13, 22], which are also common features of HCM. Moreover, Pilz et al. found that women had higher serum FFA levels than men although there were fewer women participants [22], implying that women may be at greater risk of SCD. Besides, female patients with type 2 diabetes had greater cardiac risk than males [40]. In contrast, the rate of SCD is higher among males compared to females in HCM [4]. This phenomenon may be attributed to greater LV hypertrophy in male patients. As mentioned above, sex has important influences on LV hypertrophy of HCM. And myocardial FFA metabolism may be one of the pathways.

There is considerable evidence that high FFA levels increase cardiac sympathetic activity [22, 41]. In a study of 19 healthy volunteers, Stepniakowski et al. suggested that plasma FFA enhanced vascular α-adrenergic sensitivity [42]. Furthermore, Gosmanov et al. reported that oral and intravenous fat load significantly increased SBP and heart rate in obese healthy participants [43]. In addition, parallel changes in plasma FFA levels with cardiac sympathetic/parasympathetic nervous system balance were found in type 2 diabetes patients [44]. Our study in male HCM patients showed that SBP and heart rate were significantly associated with plasma FFA levels. Although speculative, it seems likely that high FFA levels also increase cardiac sympathetic activity in male HCM patients.

Of note, men were much more likely to be smokers in our study, which may be a confounder. However, our data showed that smoking did not affect FFA levels in both men and women. Besides, statin therapy may be a major confounder. Barbarash et al. studied 210 patients with acute myocardial infarction (AMI) and reported that atorvastatin resulted in a decrease in FFA levels 12 days after AMI [45]. However, atorvastatin 40 mg/day was less efficient than 20 mg/day in their study. The decrease in FFA levels observed by Barbarash et al. may be due to relief of stress after AMI rather than atorvastatin treatment. In the present study, statins were not found to affect FFA levels in both men and women. Consistent with our data, previous studies showed that statins did not have effects on FFA levels in patients with the metabolic syndrome [46] and cardiac hypertrophy in patients with HCM [47].

Aiming at inefficient myocardial energy utilization in HCM, several drugs are introduced into the management of HCM, such as perhexiline and trimetazidine [48, 49], which can switch the heart metabolism from free fatty acid to carbohydrate utilization. Given our findings that FFA correlated characteristics of HCM only in male patients, it may be necessary to verify whether these therapeutic strategies are ineffective in female patients.

There are some limitations in our study. First, plasma FFAs were measured once and totally. Serial and classified measurements of FFAs might provide more implications and increase sensitivity for correlation. Even so, a significant correlation between plasma FFA levels and variables reflecting severity of HCM was detected in our study with large sample size using CMRI. Second, in an attempt to elucidate the role of FFA in the pathogenesis of HCM, genetic testing for HCM may be helpful. Third, in order to determine whether the relationship between FFA and HCM is causal or circumstantial, it is intriguing to investigate plasma FFA levels in mutation carriers without left ventricular hypertrophy in comparison with subjects with overt hypertrophic cardiomyopathy. Finally, this is a cross-sectional and single-center study. Considering that elevated FFA levels are shown to be an independent predictor of sudden cardiac death in patients with other heart diseases, the potential prognostic values of plasma FFA for cardiovascular events in patients with HCM are needed to be revealed in further investigation with follow-up.

## Conclusions

In patients with HCM, LVMI, LAD, SBP, and heart rate were independently associated with increasing plasma FFA levels in males, whereas not in females. These results suggest that sex may affect the pathogenesis of HCM. And these sex-related differences should be taken into account in therapeutic approaches to influence myocardial FFA metabolism in HCM.

## Abbreviations

BMI: Body mass index
CMRI: Cardiovascular magnetic resonance imaging
FFA: Free fatty acid
HCM: Hypertrophic cardiomyopathy
LAD: Left atrium diameter
LV: Left ventricular
LVEF: Left ventricular ejection fraction
LVM: Left ventricular mass
LVMI: LVM index
LVOT: Left ventricular outflow tract
MWT: Maximum wall thickness
SBP: Systolic blood pressure

## References

[1] Maron BJ, Maron MS (2015). The 20 advances that have defined contemporary hypertrophic cardiomyopathy. Trends Cardiovasc Med.

[2] Frey N, Luedde M, Katus HA (2012). Mechanisms of disease: hypertrophic cardiomyopathy. Nat Rev Cardiol.

[3] Maron BJ, Maron MS (2013). Hypertrophic cardiomyopathy. Lancet.

[4] Maron BJ, Maron MS, Semsarian C (2012). Genetics of hypertrophic cardiomyopathy after 20 years: clinical perspectives. J Am Coll Cardiol.

[5] Hensley N, Dietrich J, Nyhan D, Mitter N, Yee MS, Brady M (2015). Hypertrophic cardiomyopathy: a review. Anesth Analg.

[6] Marsiglia JD, Pereira AC (2014). Hypertrophic cardiomyopathy: how do mutations lead to disease?. Arq Bras Cardiol.

[7] Olivotto I, Maron MS, Adabag AS, Casey SA, Vargiu D, Link MS (2005). Gender-related differences in the clinical presentation and outcome of hypertrophic cardiomyopathy. J Am Coll Cardiol.

[8] Page SP, Kounas S, Syrris P, Christiansen M, Frank-Hansen R, Andersen PS (2012). Cardiac myosin binding protein-C mutations in families with hypertrophic cardiomyopathy: disease expression in relation to age, gender, and long term outcome. Circ Cardiovasc Genet.

[9] Kubo T, Kitaoka H, Okawa M, Hirota T, Hayato K, Yamasaki N (2010). Gender-specific differences in the clinical features of hypertrophic cardiomyopathy in a community-based Japanese population: results from Kochi RYOMA study. J Cardiol.

[10] Mandavia CH, Pulakat L, DeMarco V, Sowers JR (2012). Over-nutrition and metabolic cardiomyopathy. Metabolism.

[11] Vakrou S, Abraham MR (2014). Hypertrophic cardiomyopathy: a heart in need of an energy bar?. Front Physiol.

[12] Havmoeller R, Reinier K, Teodorescu C, Ahmadi N, Kwok D, Uy-Evanado A (2014). Elevated plasma free fatty acids are associated with sudden death: a prospective community-based evaluation at the time of cardiac arrest. Heart Rhythm.

[13] Opie LH (2004). The metabolic vicious cycle in heart failure. Lancet.

[14] Fukushima A, Milner K, Gupta A, Lopaschuk GD (2015). Myocardial energy substrate metabolism in heart failure: from pathways to therapeutic targets. Curr Pharm Des.

[15] Lehman JJ, Kelly DP (2002). Transcriptional activation of energy metabolic switches in the developing and hypertrophied heart. Clin Exp Pharmacol Physiol.

[16] Osorio JC, Stanley WC, Linke A, Castellari M, Diep QN, Panchal AR (2002). Impaired myocardial fatty acid oxidation and reduced protein expression of retinoid X receptor-alpha in pacing-induced heart failure. Circulation.

[17] Takeishi Y, Chiba J, Abe S, Tonooka I, Komatani A, Tomoike H (1992). Heterogeneous myocardial distribution of iodine-123 15-(p-iodophenyl)-3-R, S-methylpentadecanoic acid (BMIPP) in patients with hypertrophic cardiomyopathy. Eur J Nucl Med.

[18] Maron BJ, Verter J (1978). Disproportionate ventricular septal thickening in the developing normal human heart. Circulation.

[19] Lopaschuk GD, Jaswal JS (2010). Energy metabolic phenotype of the cardiomyocyte during development, differentiation, and postnatal maturation. J Cardiovasc Pharmacol.

[20] Reller MD, Kaplan S (1988). Hypertrophic cardiomyopathy in infants of diabetic mothers: an update. Am J Perinatol.

[21] Kadkhodayan A, Lin CH, Coggan AR, Kisrieva-Ware Z, Schechtman KB, Novak E, et al. Sex affects myocardial blood flow and fatty acid substrate metabolism in humans with nonischemic heart failure. J Nucl Cardiol. 2016. [Epub ahead of print] PubMed PMID: 27048307. 10.1007/s12350-016-0467-6 PMC5517366 27048307

[22] Pilz S, Scharnagl H, Tiran B, Wellnitz B, Seelhorst U, Boehm BO (2007). Elevated plasma free fatty acids predict sudden cardiac death: a 6.85-year follow-up of 3315 patients after coronary angiography. Eur Heart J.

[23] Mauvais-Jarvis F (2015). Sex differences in metabolic homeostasis, diabetes, and obesity. Biol Sex Differ.

[24] Chan AK, Somarouthu B, Ghoshhajra B (2014). Magnetic resonance imaging for hypertrophic cardiomyopathy update. Top Magn Reson Imaging.

[25] Elliott PM, Anastasakis A, Borger MA, Borggrefe M, Cecchi F, Charron P (2014). 2014 ESC guidelines on diagnosis and management of hypertrophic cardiomyopathy: the task force for the diagnosis and management of hypertrophic cardiomyopathy of the European Society of Cardiology (ESC). Eur Heart J.

[26] Nagueh SF, Bierig SM, Budoff MJ, Desai M, Dilsizian V, Eidem B (2011). American Society of Echocardiography clinical recommendations for multimodality cardiovascular imaging of patients with hypertrophic cardiomyopathy: endorsed by the American Society of Nuclear Cardiology, Society for Cardiovascular Magnetic Resonance, and Society of Cardiovascular Computed Tomography. J Am Soc Echocardiogr.

[27] Zhang C, Liu R, Yuan J, Cui J, Hu F, Yang W (2015). Significance and determinants of cardiac troponin I in patients with obstructive hypertrophic cardiomyopathy. Am J Cardiol.

[28] Javadpour MM, Tardiff JC, Pinz I, Ingwall JS (2003). Decreased energetics in murine hearts bearing the R92Q mutation in cardiac troponin T. J Clin Invest.

[29] Force T, Bonow RO, Houser SR, Solaro RJ, Hershberger RE, Adhikari B (2010). Research priorities in hypertrophic cardiomyopathy: report of a working group of the National Heart, Lung, and Blood Institute. Circulation.

[30] Koutsari C, Basu R, Rizza RA, Nair KS, Khosla S, Jensen MD (2011). Nonoxidative free fatty acid disposal is greater in young women than men. J Clin Endocrinol Metab.

[31] Clark CJ (1971). Carbohydrate metabolism in the isolated fetal rat heart. Am J Physiol.

[32] Amri EZ, Ailhaud G, Grimaldi PA (1994). Fatty acids as signal transducing molecules: involvement in the differentiation of preadipose to adipose cells. J Lipid Res.

[33] Way GL, Wolfe RR, Eshaghpour E, Bender RL, Jaffe RB, Ruttenberg HD (1979). The natural history of hypertrophic cardiomyopathy in infants of diabetic mothers. J Pediatr.

[34] Tanaka T, Sohmiya K, Kawamura K (1997). Is CD36 deficiency an etiology of hereditary hypertrophic cardiomyopathy?. J Mol Cell Cardiol.

[35] Tuunanen H, Kuusisto J, Toikka J, Jaaskelainen P, Marjamaki P, Peuhkurinen K (2007). Myocardial perfusion, oxidative metabolism, and free fatty acid uptake in patients with hypertrophic cardiomyopathy attributable to the Asp175Asn mutation in the alpha-tropomyosin gene: a positron emission tomography study. J Nucl Cardiol.

[36] Birch C, Behunin S, Lopez-Pier M, Danilo C, Lipovka Y, Saripalli C, et al. Sex dimorphisms of crossbridge cycling kinetics in transgenic hypertrophic cardiomyopathy mice. Am J Physiol Heart Circ Physiol. 2016;311(1):H125–36. 10.1152/ajpheart.00592.2015 PMC4967209 27199124

[37] Devanathan S, Whitehead TD, Fettig N, Gropler RJ, Nemanich S, Shoghi KI (2016). Sexual dimorphism in myocardial acylcarnitine and triglyceride metabolism. Biol Sex Differ.

[38] Arnold AP, Reue K, Eghbali M, Vilain E, Chen X, Ghahramani N (2016). The importance of having two X chromosomes. Philos Trans R Soc Lond B Biol Sci.

[39] Chen Y, Zhang Z, Hu F, Yang W, Yuan J, Cui J (2015). 17β-estradiol prevents cardiac diastolic dysfunction by stimulating mitochondrial function: a preclinical study in a mouse model of a human hypertrophic cardiomyopathy mutation. J Steroid Biochem Mol Biol.

[40] Ko DT, Wijeysundera HC, Udell JA, Vaccarino V, Austin PC, Guo H (2014). Traditional cardiovascular risk factors and the presence of obstructive coronary artery disease in men and women. Can J Cardiol.

[41] Sarafidis PA, Bakris GL (2007). Non-esterified fatty acids and blood pressure elevation: a mechanism for hypertension in subjects with obesity/insulin resistance?. J Hum Hypertens.

[42] Stepniakowski KT, Goodfriend TL, Egan BM (1995). Fatty acids enhance vascular alpha-adrenergic sensitivity. Hypertension.

[43] Gosmanov AR, Smiley DD, Robalino G, Siquiera J, Khan B, Le NA (2010). Effects of oral and intravenous fat load on blood pressure, endothelial function, sympathetic activity, and oxidative stress in obese healthy subjects. Am J Physiol Endocrinol Metab.

[44] Manzella D, Barbieri M, Rizzo MR, Ragno E, Passariello N, Gambardella A (2001). Role of free fatty acids on cardiac autonomic nervous system in noninsulin-dependent diabetic patients: effects of metabolic control. J Clin Endocrinol Metab.

[45] Barbarash O, Gruzdeva O, Uchasova E, Belik E, Dyleva Y, Karetnikova V (2015). Dose-dependent effects of atorvastatin on myocardial infarction. Drug Des Devel Ther.

[46] Huptas S, Geiss HC, Otto C, Parhofer KG (2006). Effect of atorvastatin (10 mg/day) on glucose metabolism in patients with the metabolic syndrome. Am J Cardiol.

[47] Nagueh SF, Lombardi R, Tan Y, Wang J, Willerson JT, Marian AJ (2010). Atorvastatin and cardiac hypertrophy and function in hypertrophic cardiomyopathy: a pilot study. Eur J Clin Invest.

[48] Abozguia K, Elliott P, McKenna W, Phan TT, Nallur-Shivu G, Ahmed I (2010). Metabolic modulator perhexiline corrects energy deficiency and improves exercise capacity in symptomatic hypertrophic cardiomyopathy. Circulation.

[49] Leviner DB, Hochhauser E, Arad M (2015). Inherited cardiomyopathies—novel therapies. Pharmacol Ther.

